# Modification of Polyamide 66 for a Media-Tight Hybrid Composite with Aluminum

**DOI:** 10.3390/polym15071800

**Published:** 2023-04-06

**Authors:** Fabian Lins, Christian Kahl, Jan-Christoph Zarges, Hans-Peter Heim

**Affiliations:** Institute of Material Engineering, Polymer Engineering, University of Kassel, 34125 Kassel, Germany

**Keywords:** plastic–metal hybrids, fiber orientation, X-ray microtomography, dynamic image analysis, thermal expansion exponent

## Abstract

Metal–plastic composites are becoming increasingly important in lightweight construction. As a combination, e.g., for transmission housings in automobiles, composites made of die-cast aluminum housings and Polyamide 66 are a promising material. The interface between metal and plastic and the properties of the plastic component play an important role with regard to media tightness against transmission oil. The mechanical properties of the plastic can be matched to aluminum by glass fibers and additives. In the case of fiber-reinforced plastics, the mechanical properties depend on the fiber length and their orientation. These structural properties were investigated using computer tomography and dynamic image analysis. In addition to the mechanical properties, the thermal expansion coefficient was also investigated since a strongly different coefficient of the joining partners leads to stresses in the interface. Polyamide 66 was processed with 30 wt% glass fibers to align the mechanical and thermal expansion properties to those of aluminum. In contrast to the reinforcement additives, an impact modifier to improve the toughness of the composite, and/or a calcium stearate to exert influence on the rheological behavior of the composite, were used. The combination of the glass fibers with calcium stearate in Polyamide 66 led to high stiffnesses (11,500 MPa) and strengths (200 MPa), which were closest to those of aluminum. The coefficient of thermal expansion was found to be 6.6 × 10^−6^/K for the combination of Polyamide 66 with 30 wt% glass fiber and shows a low expansion exponent compared to neat Polamid 66. It was detected that the use of an impact modifier led to less orientated fibers along the injection direction, which resulted in lower modulus and strength in terms of mechanical properties.

## 1. Introduction

The combination of two materials makes it possible to combine the positive properties of both partners [1]. Metal–plastic hybrid composites are therefore a promising way to reduce the weight of, for example, automobiles and, thus, CO_2_ emissions [2]. Due to their high mechanical properties, fiber-reinforced plastics and aluminum, with their low densities, play a major role in this material combination [3].

In-mold technology has become established for the production of metal–plastic composites. A metal insert is placed in an injection mold and the plastic is injected with an injection molding machine [4,5]. One challenge in combining the two materials is the interface between the metal and the plastic, as these have different mechanical and thermal characteristic values [3]. When metal–plastic joints are produced by injection molding, the quality of the finished part significantly depends on the adhesion of the composite. The metal part is often pretreated by chemical etching or sandblasting to generate a rough surface [6]. The rough surface creates strong bonding by mechanically anchoring the melt to the pretreated surface [7].

Especially for the automotive industry, a strong bond between metal and plastic is essential. The substitution of non-load-bearing areas in a die-cast housing with plastic must also provide tightness against media such as transmission oil on the interface between plastic and metal. 

The influence of additives, such as an impact modifier or a lubricant, has already been demonstrated in combination with Polyamide 6 [8]. The viscosity of the polymer can be influenced by the impact modifier and the calcium stearate. The mechanical properties of the polyamide are increased and brought as close as possible to those of aluminum by the addition of glass fibers. This study investigated to what extent the impact modifier and calcium stearate influence the fiber length and the fiber orientation.

For a media-tight bond between Polyamide 66 and aluminum, the interface plays an important role. In order to bond the two joining partners as strongly as possible, the surface is roughened and/or an coupling agent is used. Ultimately, however, the failure of the bond is caused by stress peaks caused by brittle properties or different expansion coefficients. In this project, it will later be investigated whether a less brittle material can enable a long-lasting, tight connection between the joining partners, which can guarantee tightness even with small movements and vibrations due to higher ductility. For this reason, the fiber-reinforced material will be provided with additives such as a lubricant and/or an impact modifier and investigated with regard to the mechanical properties and the thermal expansion coefficient. This study can be seen as preliminary work for the further investigation of composite Polyamide 66 and aluminum. 

The examination of glass fibers in a selected volume with regard to fiber length and fiber orientation is possible through 3-dimensional analysis by X-ray microtomography [9,10]. Due to the high density of the glass fiber of 2.6 g/cm^3^, it can be easily separated from the polyamide with a density of 1.15 g/cm^3^. In 2015, Nguyen Thi et al. investigated glass fibers in Polyamide 6 using X-ray microtomography and were able to visualize and evaluate the fibers in terms of length and their orientation at different locations within a test specimen [11].

In addition to the mechanical properties, the coefficient of thermal expansion plays a major role with regard to the bond strength. If the coefficient of thermal expansion of the two components is very different, the different behavior during cooling has a negative effect on the bond strength. A similar behavior of the two joining partners means a low interfacial stress. For this reason, fiber-reinforced polymers are used for applications in joining metal and plastic in order to obtain high dimensional stability under thermal influences, and during cooling after injection molding [12,13]. Heckert et al. were able to show that the coefficient of thermal expansion decreased as the weight content of fibers in a fiber-reinforced polymer increased. The coefficient of thermal expansion for Polyamide 6 is 85 × 10^−6^/K and could be reduced to 50 × 10^−6^/K by adding 60 wt% glass fiber [14].

## 2. Materials and Methods

### 2.1. Materials

The base polymer used for this study was Polyamide 66 (PA66) from BASF (Ludwigshafen, Germany). The grade Ultramid A27E has a density of 1.14 g/cm^3^ and is a special grade for compounding. Before processing, the polyamide was dried in a Dry Jet Easy Dryer from TORO-Systems (Igensdorf, Germany) for at least 4 h at 80 °C to achieve a moisture content of max 0.2%. The PA66 was used as a non-colored grade.

To reinforce the polyamide, glass fibers were added as short fibers with a content of 30 wt% in the compounding process. The glass fibers of type CS 7928 were purchased from Lanxess Germany GmbH (Cologne, Germany). The fibers had an initial length of 4.5 mm and a fiber diameter of 11 µm. A sizing on the surface of this fiber type promised good adhesion to polyamides.

The use of an impact modifier leads to an increase in the toughness of a plastic and was, therefore, used here to modify the properties. An impact modifier from Kraton Polymers LLC (Houston, TX, USA) was added to the polyamide in two components. One component of the modifier was type FG1901, which consists of a linear triblock copolymer based on styrene and ethylene/butylene with a polystyrene content of 30%. This component was added with a weight content of 8%. The second component was type G1657, which is a linear triblock copolymer based on styrene and ethylene/butylene with a polystyrene content of 13%. This component was added with a weight content of 12%. Both components had a density of 0.9 g/cm^3^. The weight content of the impact modifier was 20% in total. The components and their weight contents were selected according to a recommendation of the manufacturer [15].

The lubricant calcium stearate from the Faci Group (Genova, Italy) was also used to reduce the viscosity of the polyamide melt. The salt is also used as a lubricant in pharmaceutical products and as a lubricant in the paper and metal processing industries. It has a melting point of 150 °C and a density of 2.6 g/cm^3^. The stearate was used with a weight content of 0.1%. After consultation with the manufacturer, it was advised that a higher concentration would have a negative effect on the adhesion properties between the plastic compound and the aluminum.

### 2.2. Compounding

The compounds were produced on a co-rotating twin-screw extruder from Leistritz (Nuremberg, Germany), type ZSE18 HPe. The barrel has a diameter of 18 mm and an L/D ratio of 40. The extruder is equipped with a screw configuration that has a high proportion of conveying elements. This configuration is intended to generate lower shear energy in the melt to prevent severe fiber shortening. The impact modifier and the lubricant were mixed into the PA66 granules and added to the compounding process by a gravimetric feeder from Brabender (Duisburg, Germany). After melting of the granules, the glass fibers were also added to the process by a gravimetric feeder. The melt temperature during compounding was 280 °C and the screw speed was 200 rpm. The extruded material strand was cooled with compressed air after exiting the die and then cut into granules with a length of 3 mm in a pelletizer of the type Scheer SGS 25-E (Grossostheim, Aschaffenburg, Germany). The material composition of the PA66, the additives, and the glass fibers can be seen in Table 1.

In the following table and in the results section, the compounds are described with the abbreviations glass fiber (GF), impact modifier (IM), and calcium stearate (CaSt).

### 2.3. Injection Molding

The specimens, according to DIN EN ISO 527 Type 1A, were injection-molded for mechanical characterization and X-ray microtomography (µ-CT). An injection molding machine from Arburg (Loßburg, Germany) type 320C Golden Edition was used for this purpose. The machine has a clamping force of 500 kN. The temperature along the screw was set to 290 °C and the injection speed was set to 16 cm^3^/s. The mold temperature was set to 80 °C. All compounds were dried at 80 °C for at least 4 h before processing.

### 2.4. Tensile Testing

Tensile tests to DIN EN ISO 527 were performed on a universal testing machine, type Z010 from Zwick Roell (Ulm, Germany). The tests were performed at a speed of 5 mm/min and the Young’s modulus, tensile strength, and elongation at break were evaluated.

### 2.5. Dynamic Image Analysis (DIA)

For dynamic image analysis, the fibers were separated from the matrix material by ashing the samples at 600 °C for 6 h. The system QICPIC/R06 (Sympatec, Clausthal-Zellerfeld, Germany) with a MIXCEL wet dispersion unit was used to measure the fiber length distribution. The fibers were dispersed in isopropanol. The images were acquired at a rate of 175 Hz and a resolution of 4.2 MP. A cuvette with a width of 0.5 mm was used and the fibers were measured with an M5 objective. This objective records fibers with a length of 1.8 µm to 3700 µm. For each sample, 3 measurements of 60 s were performed. Approximately 10,000 fibers were measured for each measurement. The fibers were evaluated with the LEFI (length of fiber) module, which measures the shortest distance between the endpoints of the fiber.

### 2.6. X-ray Microtomography

**Figure 1:**
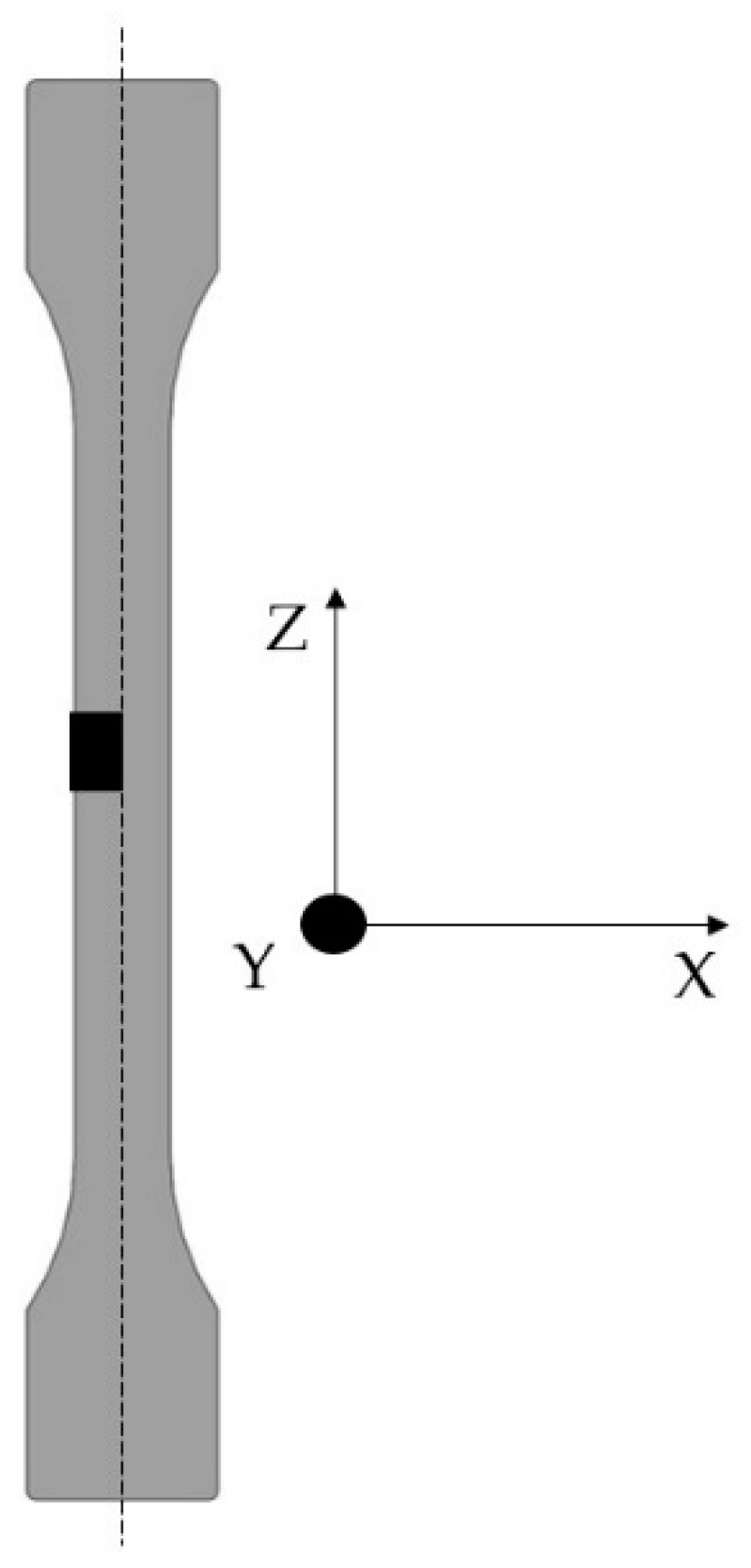
Evaluated volume of tensile specimen according to DIN EN ISO 527 for orientation and length of glass fiber.

A volume shown in Figure 1 was examined by X-ray microtomography to compare the fiber length in the volume with the fiber lengths from DIA and to examine the fiber orientation along the flow direction. For this purpose, a Zeiss Xradia Versa 520 (Oberkochen, Germany) was used. The measurements were performed at a voltage of 80 kV and a current of 87.2 µA. A volume of size 4 mm × 5 mm × 12 mm from the middle of a tensile specimen was recorded, as shown in Figure 1, with a 5279 µm field of view and a pixel size of 5.2 µm. Avizo 9.4 software was used to compose the 1600 taken images and a volume of 5 mm × 2 mm × 2 mm was used to display the optical fibers and to evaluate them in terms of orientation and length. The flow direction during mold filling corresponds to the z-axis. The deviation of the fiber from the z-axis is represented with an angle theta (θ). Thus, a fiber with an angle θ = 0° is oriented along the z-axis and an angle of θ = 90° shows that the fiber is oriented orthogonally to the z-axis. About 60,000 fibers were evaluated in the volume of the specimen. Fiber length in X-ray microtomography was evaluated to provide a reference for the results of the DIA.

### 2.7. Dynamic Mechanical Analysis

The coefficient of thermal expansion was determined by dynamic mechanical analysis (DMA) with variation in the temperature and a constant load. A Q 800 module from TA Instruments (Hüllhorst, Germany) was used for this purpose. The specimens for the measurements had dimensions of 30 mm × 10 mm × 4 mm and the clamp distance was 20 mm. The coefficient of expansion was measured in a temperature range from 25 to 100 °C and the heating rate was 3 °C/min. A constant load of 10 N was applied to the specimen. For the evaluation, the average coefficient of linear expansion ($\bar{\alpha }$) was determined using the following formula:

(1) $$\bar{\alpha }=dL/(dT*L_{0})$$

**Figure 2:**
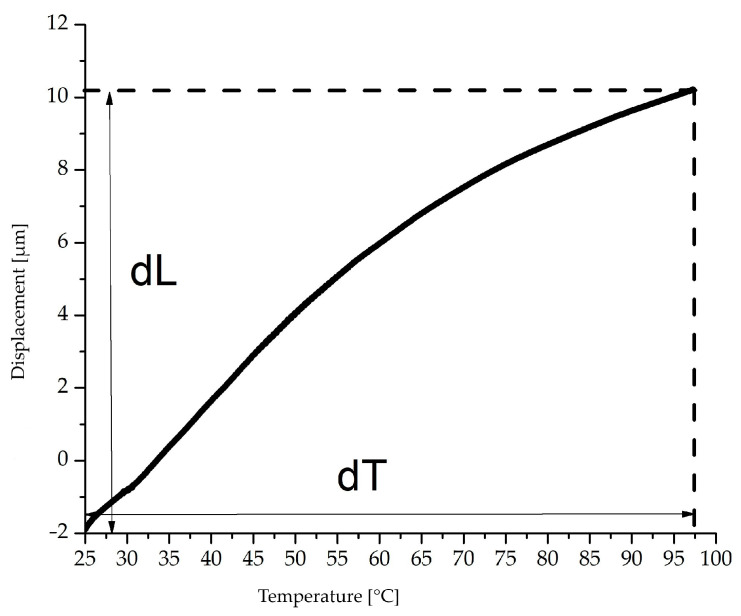
Displacement and temperature course of DMA measurements with constant load at 10 N.

The mean coefficient of thermal expansion is represented by $\bar{\alpha }$ taken from the first and last measuring points (dL) according to Figure 2. This is divided by the temperature difference (dT) and the original length (L_0_). Three specimens were tested for each compound.

## 3. Results and Discussion

### 3.1. Tensile Test

For the evaluation of the results, tensile test specimens were first manufactured according to DIN EN ISO 527 and tested in tensile tests. The use of various additives, such as an impact modifier or a lubricant, changes the viscosity of the melt and the flow properties. In this study, the mechanical properties as well as the glass fiber orientation and the length of the fibers in the test specimens were demonstrated on Polyamide 66 with the use of the individual additives and considering the interaction of both additives.

**Figure 3:**
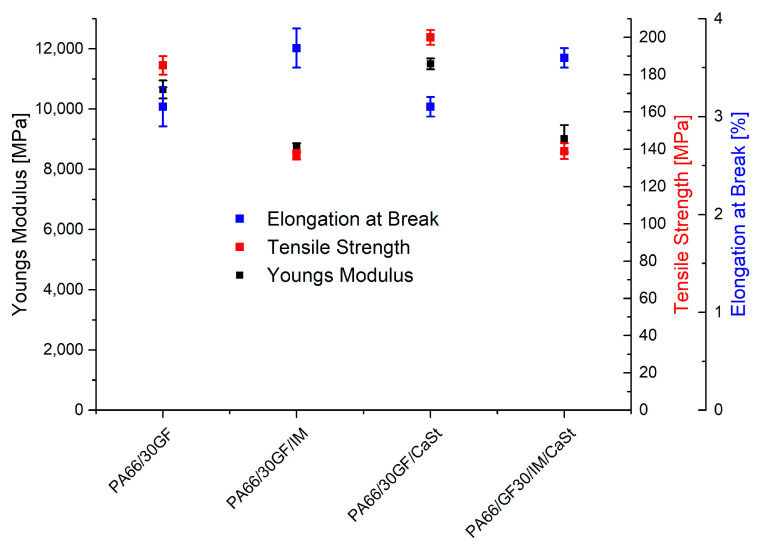
Results of tensile test with PA66/30GF-based specimen.

Figure 3 shows the results of the tensile tests. An increase in the elongation at break was clearly visible between the samples PA66/30GF and PA66/30GF/IM. By adding a weight fraction of 20 wt% of the impact modifier to the compound PA66/30GF, the elongation at break increased from 3.1% to 3.7%, while the tensile strength decreased from 185 MPa to 137 MPa. On the one hand, this may have been due to the change in the chemical structure caused by the impact modifier. The modifier largely consisted of styrene and formed a two-phase mixture due to its incompatibility with polyamide [16]. On the other hand, it is possible that the impact modifier changed the bond of the fiber to the matrix. If the fiber is in contact with the impact modifier, the adhesion to it is worse than to the PA66. As a result, the reinforcing effect is reduced due to lower force transmission.

The elongation at break of the PA66/30GF/IM/CaSt compound was also similar to that of the compound PA66/30GF/IM, accompanied by a reduction in tensile strength. The use of both additives was, therefore, dominated by the high degree of toughness of the impact modifier. The compounds contained 0.1% by weight of CaSt. Such a small amount was sufficient to have an influence on the flow properties of the polyamide melt. In terms of mechanical properties, a slight increase in tensile strength was seen for the compound PA66/30GF/CaSt at 200 MPa compared to the PA66/30GF at 185 MPa. The Young’s modulus was also increased from 10,650 MPa to 11,500 MPa by using calcium stearate. The flow properties of the compound with calcium stearate led to better orientation of the fiber to the flow direction, which was reflected in enhanced mechanical properties [17].

For a media-tight composite of aluminum and PA66, the approach used was to make the reinforced PA66 tougher by means of additives so that the media-tight composite could be maintained even in the case of vibrations and small movements in the interface. For the later tests of the composite, variation in the mechanical properties was, therefore, possible with the additives used here. The use of an impact modifier was able to increase the toughness of the plastic even with a fiber weight content of 30%.

### 3.2. Dynamic Image Analysis (DIA)

**Figure 4:**
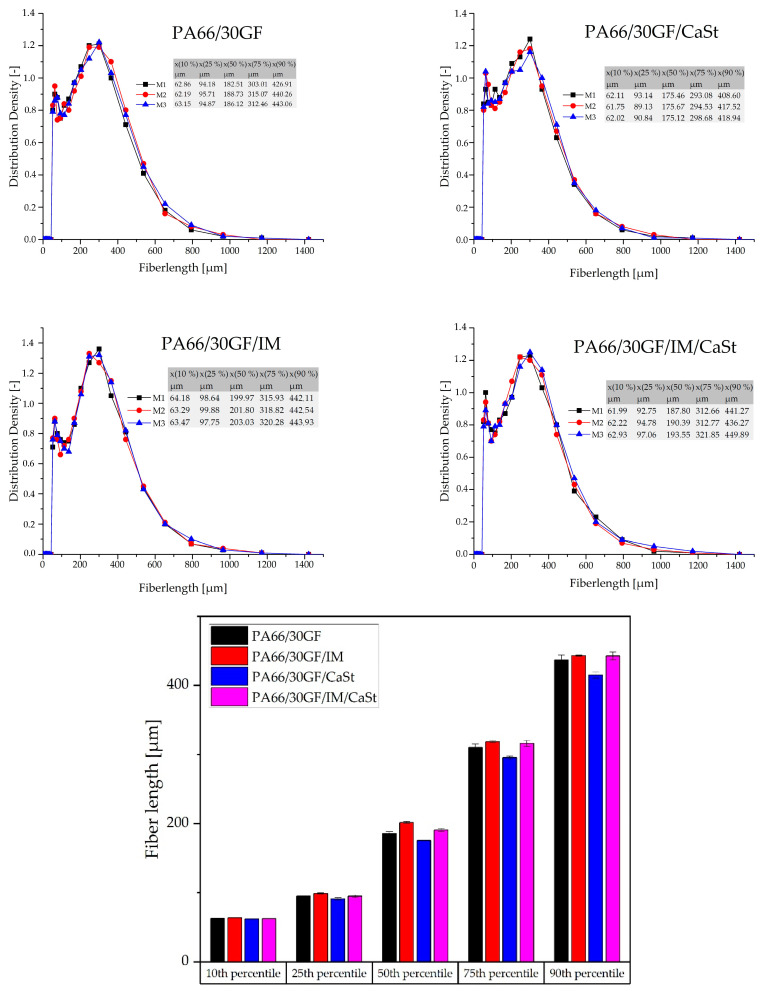
Results of the distribution density of measured fiber length from dynamic image analysis.

The fibers detached from the matrix by ashing were measured by DIA to compare the results with the fiber lengths measured from µ-CT analysis. The results of the DIA are shown in the diagrams of Figure 4. Overall, it can be seen that the fibers based on the results of DIA were longer than the fiber lengths from the X-ray microtomography. This was evident at the 75th and 90th percentiles. For the compound PA66/30GF, the 75th percentile was 260 µm when evaluated by X-ray microtomography and approximately 310 µm in the DIA. The results of the other measurements with IM or CaSt were similar. In the evaluation of the DIA results, as well as in the evaluation by the X-ray microtomography, no great change in fiber length was seen. The compound PA66/30GF/CaSt only showed slightly shorter fibers, especially at the high percentiles (75th and 90th), compared to the PA66/30GF/IM/CaSt and PA66/30GF/IM compounds. This can be explained by a decrease in the viscosity [11]. This resulted in more fiber breakage and, thus, in shorter fiber lengths.

### 3.3. X-ray Microtomography

The investigated volume from a tensile specimen included the edge region as well as the center of the specimen in order to investigate all regions with respect to orientation and fiber length.

**Figure 5:**
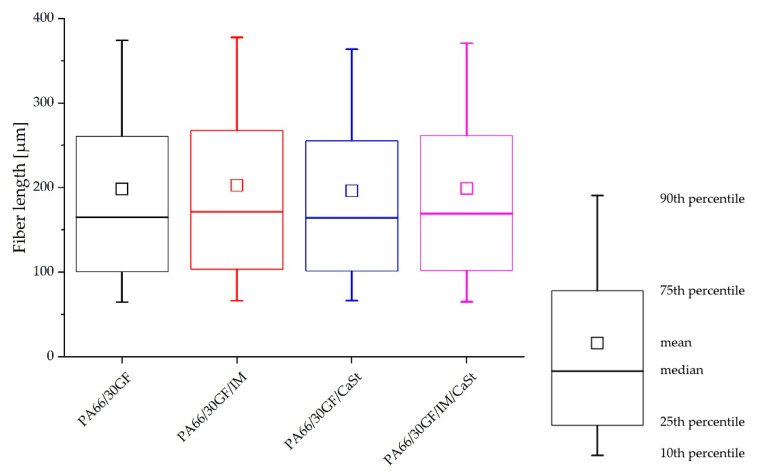
Fiber length in tensile specimen shown in boxplots from 90th percentile to 10th percentile.

Figure 5 shows the fiber lengths determined by X-ray microtomography. The differences between the four compounds were very small. The results visualized as boxplots showed a slight fiber shortening in the compound PA66/30GF/CaSt. In particular, a difference was observed at the 75th and the 90th percentiles. The differences, especially in the long fibers of the 75th and 90th percentiles, showed that the use of an impact modifier influenced the process-induced shortening of the fiber length. In this compound, the fibers were the shortest. On the other hand, the compounds with impact modifier PA66/30GF/IM and PA66/30GF/IM/CaSt had longer fibers, especially at the 75th and 90th percentiles, due to the tough properties of the impact modifier. Fiber shortening was less for these compounds.

The figure shows that a difference in fiber length along all compounds was observed in the higher values of the percentiles. At the 10% and 25% percentiles, no difference in fiber length was visible.

In addition to the fiber length, the fiber orientation was investigated since the fiber orientation also has a strong influence on the mechanical properties. In Figure 1, the z-axis specifies the flow direction. The orientation of a fiber is described by an axis of its cylindrical shape. The deviation of this axis from the flow direction is described by the angle theta (θ). The smaller the angle theta, the better the fiber is oriented in the flow direction [18].

**Figure 6:**
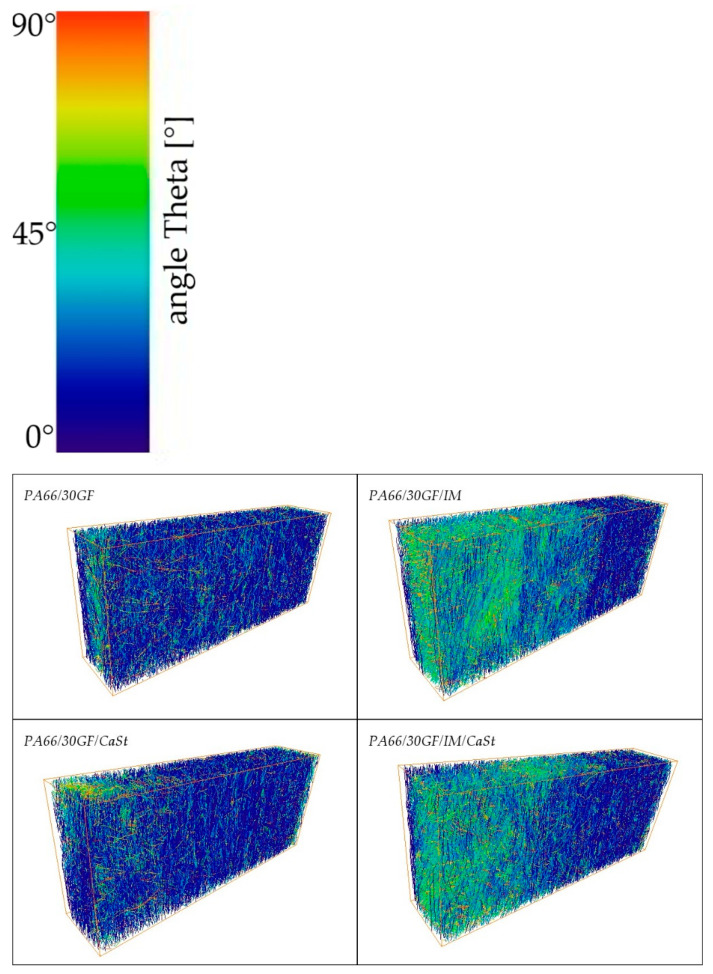
Fiber orientation of different PA66 compositions.

The orientation of the fibers can be seen in Figure 6, in which the fibers are colored according to their orientation. Blue shows the fibers well-aligned to the z-axis and those red-colored are the fibers with an orientation orthogonal to the flow direction. The figure shows a volume that was separated from a tensile specimen and examined according to Figure 1. In all four images in Figure 6, a very good orientation of the fibers can be seen in the edge region. This can be explained by the swelling flow during injection molding and has already been reported in numerous publications. In the volumes from specimens without an impact modifier, good orientation of the blue colored fibers can be seen over the whole examined area. In the case of the specimens from PA66/30GF/IM and PA66/30GF/IM/CaSt, it can be seen that the fibers were less oriented to the flow direction in the center of the specimen than in the edge region. A fiber with an orientation perpendicular to the flow direction absorbed less stress than a fiber with an orientation in the flow direction since the flow direction also corresponds to the loading direction in the tensile test. The orientation to the flow direction decreased towards the center for the samples mentioned.

**Figure 7:**
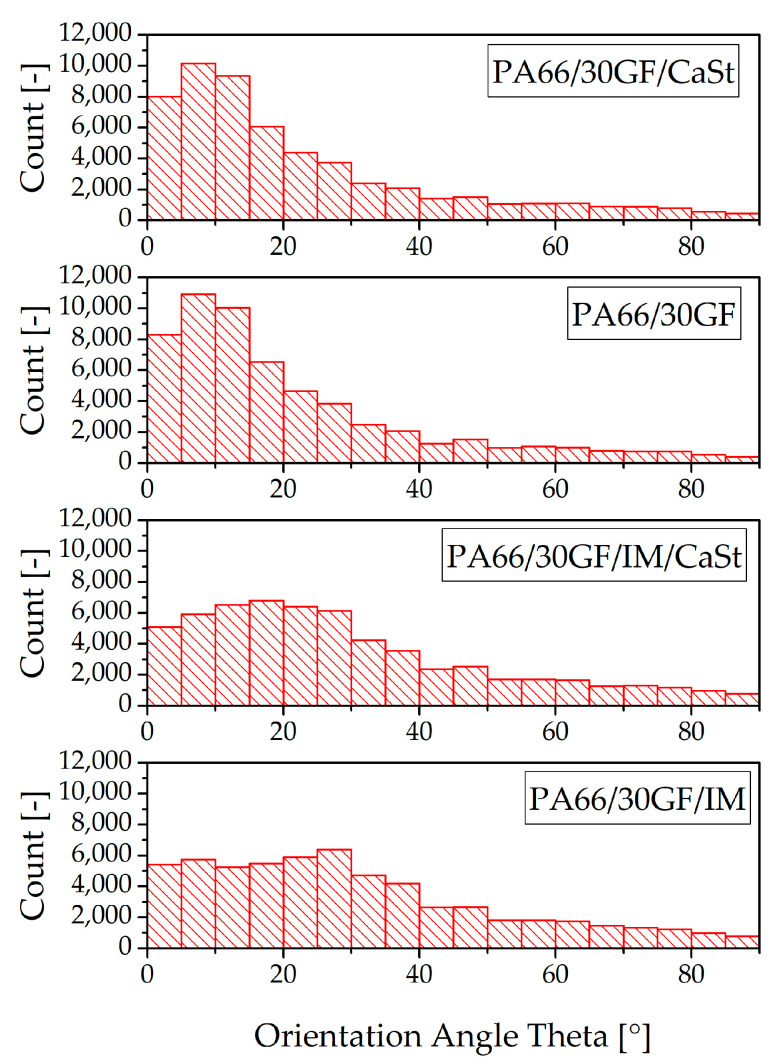
Fiber orientation in tensile specimen shown from 0° to 90°.

The fiber orientation is also illustrated quantitatively in bar graphs in Figure 7. The fibers oriented to the flow direction are shown on the left of the horizontal axis in these diagrams; the perpendicular oriented fibers are on the right. It can also be seen that a high number of fibers in the specimens without impact modifier were oriented along the flow direction. With this orientation, the fibers can absorb the forces under a tensile load in the z-direction (Figure 1) much better than fibers that are oriented perpendicular to the flow direction. The bar graphs also show that a smaller number of fibers were oriented along the flow direction in the specimens with impact modifier. This is also confirmed by the pictures in Figure 6.

The fiber orientation of the compounds with and without impact modifier also provides an explanation for the results of the tensile test in Figure 3. Here, the compounds with impact modifier showed lower stiffness and strength than the compounds without impact modifier. This was due, on the one hand, to the lower number of fibers oriented to the loading direction, and, on the other hand, to the toughness of the impact modifier. The mechanical properties of differently oriented fibers had previously been investigated by Zarges et al. In their study, specimens with fibers oriented along the direction of loading were also observed to be able to absorb a higher force than specimens with a fiber orientation transverse to the direction of loading [19].

### 3.4. Dynamic Mechanical Analysis

**Figure 8:**
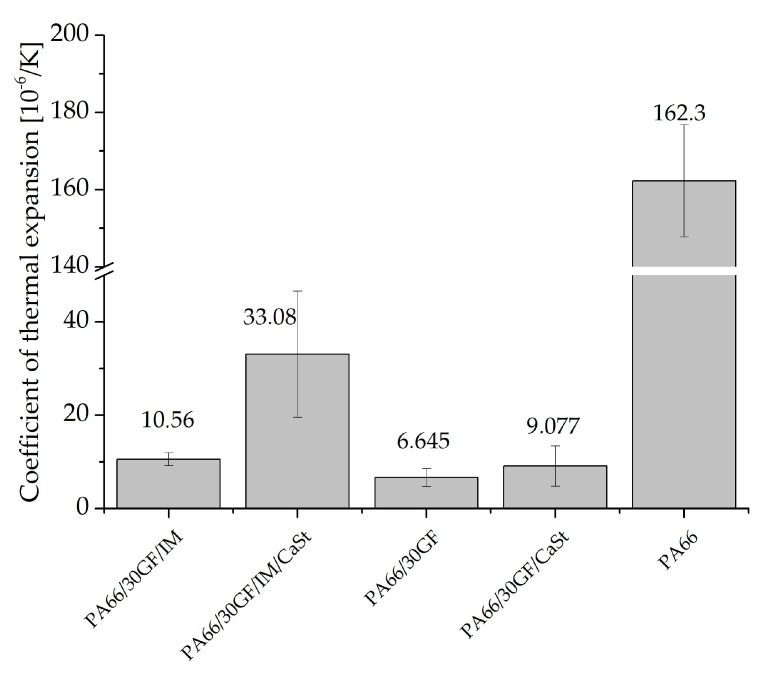
Thermal expansion exponent of compounds.

The thermal expansion of the two joining partners is of great importance after processing by injection molding. Plastic has a significantly higher coefficient of thermal expansion than aluminum. This difference results in stresses in the interface between the two joining partners, which, in turn, had a negative effect on adhesion, and, as a result, on the tightness at the interface between the plastic and the metal. For this reason, the coefficient of thermal expansion of the plastic should be brought into line with that of the aluminum [12,14,20]. Figure 8 shows the coefficients of expansion of the modified PA66, and, in comparison, the coefficient of expansion of neat PA66. In the temperature range from 25 °C to 100 °C, aluminum has a very low thermal expansion coefficient compared with plastics [21]. The coefficient can be reduced by fiber reinforcement with GF [12,20]. It can be seen that the compound PA66/30GF achieved the lowest coefficient of thermal expansion with 6.6 × 10^−6^/K. The specimens for the determination of the coefficient of thermal expansion were prepared in the same way as for the tensile tests for the injection-molded specimens according to DIN EN ISO 527. The fiber orientation in these tests was, therefore, exactly as shown in Figure 7. The addition of additives to the compound PA66/30GF led to an increase in the coefficient of expansion. As expected, the neat PA66 had the highest coefficient of thermal expansion.

## 4. Conclusions

In this study, specimens were manufactured to investigate the tensile and thermal properties of PA66 reinforced with 30 wt% glass fibers. In addition to glass fibers, an impact modifier and/or a lubricant was added to vary the properties for investigation of a media-tight plastic-metal bond. To explain the mechanical results, a volume of a specimen was characterized by X-ray microtomography and the fibers were examined in terms of length and orientation. In addition, the coefficient of thermal expansion was investigated to compare the expansion of the modified PA66 with aluminum. The following conclusions can be drawn:

- The addition of an impact modifier to GF-reinforced PA66 resulted in a higher elongation at break and a reduction in the strength and stiffness of the compound. The addition of calcium stearate led to a small increase in stiffness and strength, which was closest to the properties of aluminum.
- The fiber length measured with X-ray microtomography was only very slightly affected by the addition of the additives. In the case of the short fibers with a length of approx. 100 µm, no influence of the additives could be detected. For longer fibers of 250 µm to 400 µm, the addition of an impact modifier had a minor effect on increase in fiber length. The results of DIA produced similar results to those of X-ray microtomography. The fiber length was slightly higher in the DIA results. In summary, no significant change in fiber length due to the addition of the additives used in this study was responsible for changes in the mechanical properties.
- The impact modifier had an influence on the fiber orientation. Towards the center of specimens with impact modifier, the fibers were oriented in parallel less than in specimens without impact modifier. At the edge region, the fibers were well oriented along the flow direction due to the swell flow. The fiber orientation to the flow direction showed a clear dependence on the mechanical properties. Fibers oriented to the direction of flow showed higher strength and stiffness in the composite than fibers whose orientation deviated from the direction of flow.
- The use of individual additives led to an increase in the coefficient of thermal expansion. As a result, the very low coefficient of thermal expansion of PA66/30GF (6.6 × 10^−6^/K) was able to be brought into line with the very low coefficient of aluminum, which led to low stresses in the interface and contributed to a media-tight bond. For low stresses in the interface between aluminum and GF reinforced PA66, the addition of the additives used here was counterproductive as they increased the thermal expansion coefficient.

## Figures and Tables

**Table 1:** Compositions of charges produced by compounding.

	PA66	GF	Impact Modifier		Calcium Stearate
			Kraton FG1901	Kraton G1657	CaSt
	wt%	wt%	wt%	wt%	wt%
PA66/30GF	70	30	-	-	-
PA66/30GF/IM	50	30	8	12	-
PA66/30GF/CaSt	69.9	30	-	-	0.1
PA66/30GF/IM/CaSt	49.9	30	8	12	0.1

## Data Availability

Not applicable.
